# Supplementary material for: Reemergence of pathogenic, autoantibody-producing B cell clones in myasthenia gravis following B cell depletion therapy
Source: Acta Neuropathol Commun. 2022 Oct 28;10:154. doi: 10.1186/s40478-022-01454-0 (PMC9617453; doi:10.1186/s40478-022-01454-0)
Supplement: Supplementary file 1 — Additional file 1: Supplementary figures and tables. [file 40478_2022_1454_MOESM1_ESM.pdf]

Supplementary Information

Reemergence of pathogenic, autoantibody-producing B cell clones in myasthenia gravis following B cell depletion therapy

**Supplement Figure 1**	Validation of a fluorescently labeled MuSK ectodomain reagent to identify MuSK autoantibody-expressing cells.
**Supplement Figure 2**	MuSK flow cytometry gating strategy.
**Supplement Figure 3**	Cell-based assay contour plots showing dilution series of mAbs 2E6 and 6C6.
**Supplement Figure 4**	Staining murine neuromuscular junctions with MuSK mAbs 2E6 and 6C6.
**Supplement Figure 5**	Binding properties of unmutated common ancestors from MuSK mAbs 2E6 and 6C6.
**Supplement Figure 6**	Polyreactivity and self-reactivity of MuSK mAbs.
**Supplement Figure 7**	Somatic mutation frequency and isotype distribution of B cells examined with single cell transcriptional profiling.
**Supplement Figure 8**	Distribution of IgG4 expression among B cells clusters identified by single cell transcriptional profiling.
**Supplement Figure 9**	Distance-to-nearest plots used to identify the threshold required for assigning clonal members in the BCR sequencing data.
**Supplement Table 1**	Study subject clinical, laboratory, and demographic data.
**Supplement Table 2**	Radioimmunoassay-based testing of the 2E6 and 6C6 mAbs.
**Supplement Table 3**	Characteristics and analysis status of serial samples from patients MuSK MG-1 and MuSK MG-4.
**Supplement Table 4**	Counts of reconstructed V(D)J sequences by isotype and clones from sequencing of bulk BCR repertoires and 10x.

Supplement Figure 1

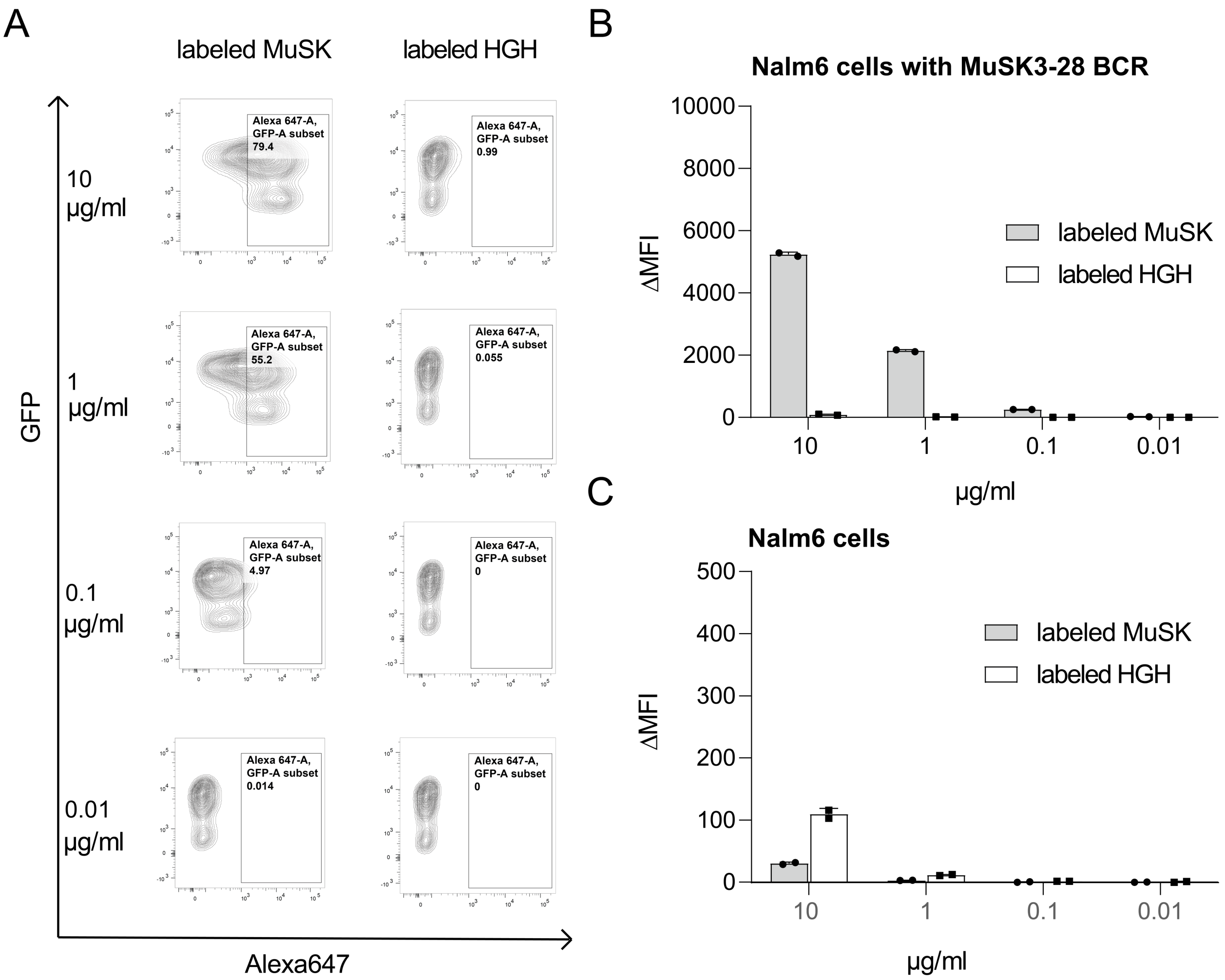

Supplement Figure 1. Validation of a fluorescently labeled MuSK ectodomain reagent to identify MuSK autoantibody-expressing cells. A monomeric fluorescently labeled (Alexa Fluor 647) MuSK ectodomain was tested for use in labeling Nalm6-GFP cells, which were engineered to express a B cell receptor (BCR) derived from a MuSK autoantibody (human recombinant mAb MuSK 3-28). Labeled human growth hormone (hGH) was used as negative control. **(a)** Representative flow cytometry contour plots are shown for MuSK and hGH binding to Nalm6-GFP cells expressing a MuSK3-28 BCR. The y-axis represents GFP fluorescence intensity. The x-axis represents Alexa Fluor 647 fluorescence intensity, which corresponds to antigen bound by the BCR. Hence, cells binding to antigen labeled with Alexa Fluor 647 are in the right quadrants. The plots show testing with an antigen (MuSK or hGH) concentration of 10, 1, 0.1 and 0.01 µg/ml. **(b, c)** Binding to antigen was tested with Nalm6-GFP cells expressing the MuSK 3-28 BCR **(b)** or unmodified Nalm6-GFP cells **(c)**. Antigen binding was tested at 10, 1, 0.1 and 0.01 µg/ml. The ΔMFI was calculated by subtracting the signal acquired from testing cells with antigen added from the signal acquired from cells with no antigen added. Each data point represents the mean of value from two independent experiments. Bars or symbols represent means and error bars SDs.

Supplement Figure 2

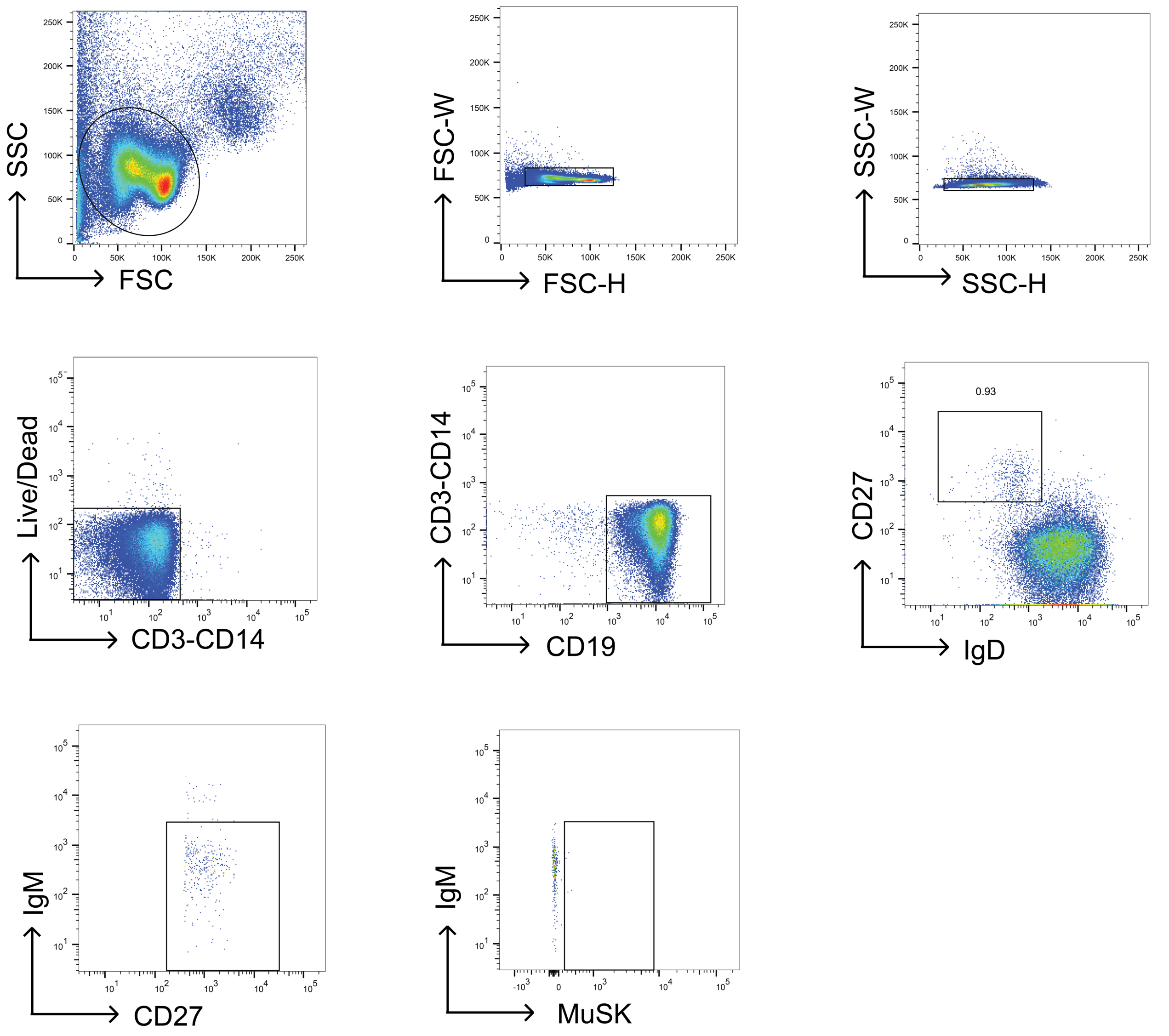

Supplement Figure 2. Flow cytometry gating strategy for isolation of MuSK-specific B cells. A representative example of the gating strategy featuring the fluorescently labeled MuSK ectodomain reagent is shown. After B cell enrichment using negative selection beads, single cells were gated using the forward (FSC) and side (SSC) scatter. Dead cells were excluded, then $CD3^{neg}$ $CD14^{neg}$ $CD19^{+}$ IgD^{neg} $CD27^{+}$ IgM^{neg} MuSK-reagent^{+} cells were single cell sorted for subsequent B cell culture and expansion.

Supplement Figure 3

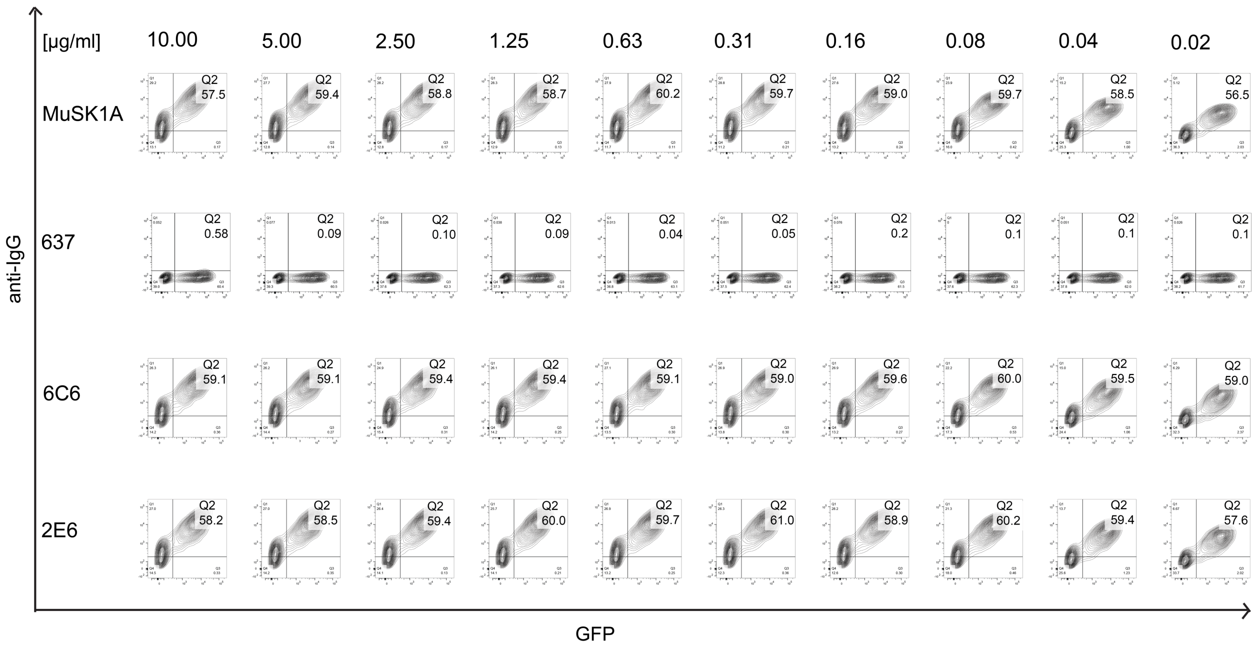

Supplement Figure 3. Cell-based assay contour plots showing dilution series of mAbs 2E6 and 6C6. Binding to MuSK was tested over a series of ten two-fold dilutions of each mAb ranging from 10-0.02 µg/ml. The x-axis represents GFP fluorescence intensity and, consequently, the fraction of HEK cells transfected with MuSK. The y-axis represents Alexa Fluor 647 fluorescence intensity, which corresponds to the secondary anti–human IgG Fc antibody binding and, consequently, primary antibody binding to MuSK. Hence, transfected cells are located in the right quadrants and cells with MuSK antibody binding in the upper quadrants. The MuSK-specific human mAb MuSK1A was used as a positive control and the AChR-specific human mAb 637 used as a negative control.

Supplement Figure 4

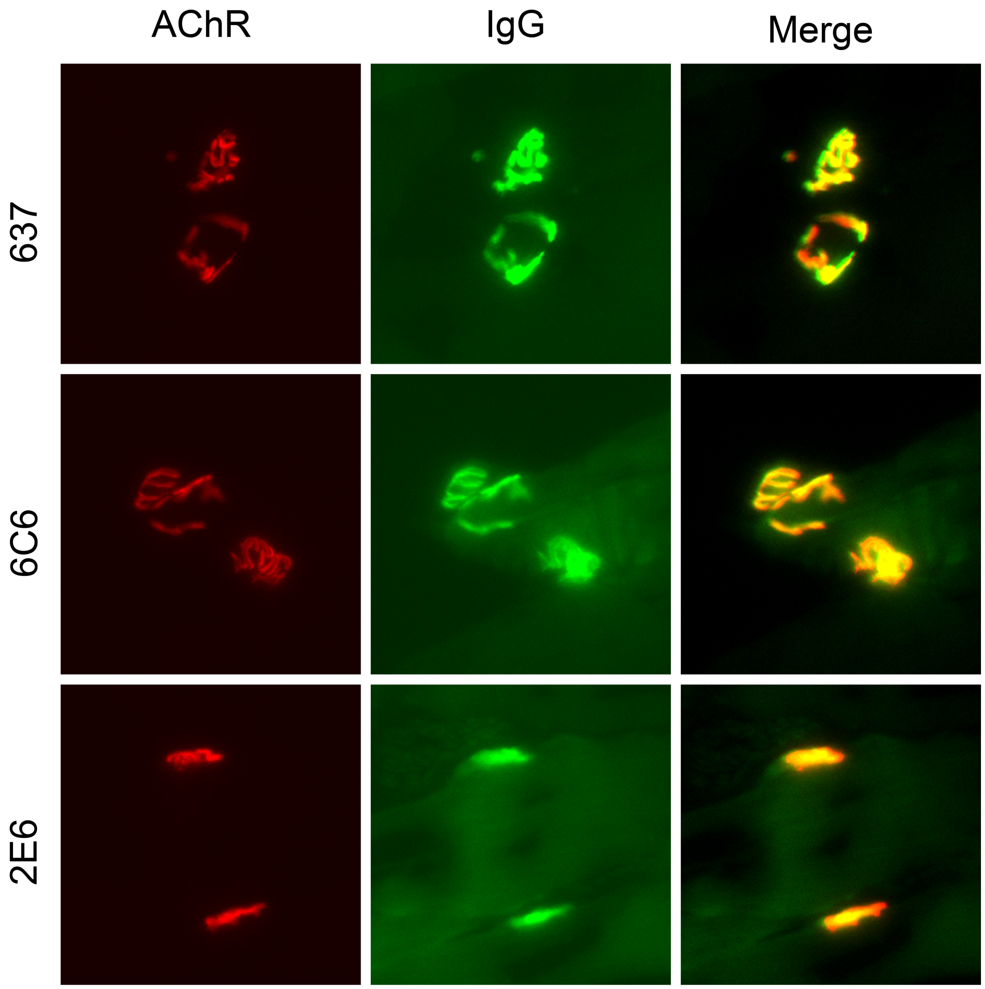

Supplement Figure 4. Staining murine neuromuscular junctions with MuSK mAbs 2E6 and 6C6. Immunofluorescent staining of mouse neuromuscular junctions (NMJ). Tibialis anterior muscles were cut longitudinally in cryosections and fixed with PFA. AChRs were stained with Alexa Fluor 648 α-bungarotoxin (shown in red). The mAb 637 was used a positive control, to identify the location of the AChR. Binding of mAbs was detected with goat anti–human IgG Alexa Fluor 488 (IgG, shown in green).

Supplement Figure 5

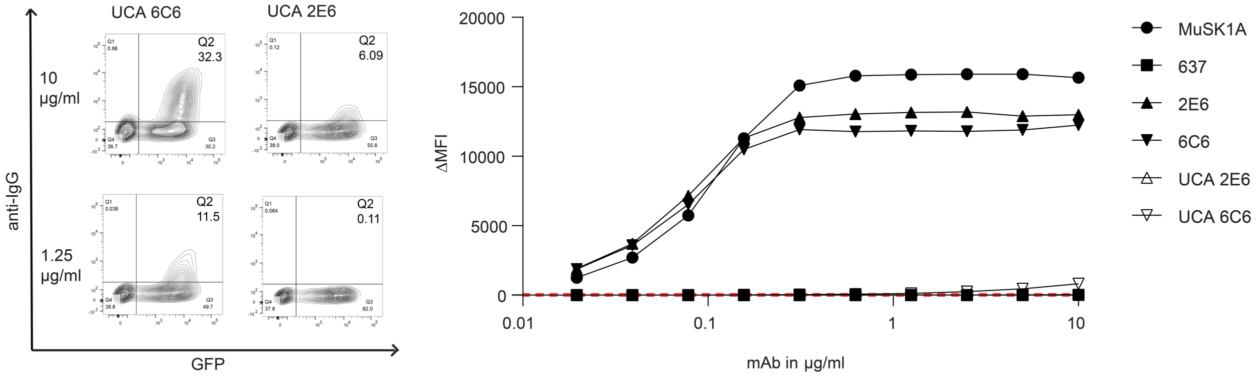

Supplement Figure 5. Binding properties of unmutated common ancestors from MuSK mAbs 2E6 and 6C6. Representative cell-based assay (CBA) contour plots are shown **(left)** for the unmutated common ancestors (UCA) of 2E6 and 6C6. The x-axis represents GFP fluorescence intensity and, consequently, the fraction of HEK cells transfected with MuSK. The y-axis represents Alexa Fluor 647 fluorescence intensity, which corresponds to secondary anti–human IgG Fc antibody binding and, consequently, primary antibody binding to MuSK. Hence, transfected cells are located in the right quadrants and cells with MuSK antibody binding in the upper quadrants. The plots show testing with a mAb concentrations of 10 and 1.25 µg/ml. Binding to MuSK was tested over a series of ten two-fold dilutions of each mAb ranging from 10-0.02 µg/ml **(right)**. The MuSK1A mAb was used as the positive control and AChR-specific mAb 637 as the negative control. The ∆MFI was calculated by subtracting the signal acquired by testing non-transfected cells from the signal acquired by testing transfected cells. Each data point represents the mean value from three independent experiments. Symbols represent means and error bars SDs. Values greater than the mean + 4SD of the negative control mAb at 1.25 µg/ml (indicated by the horizontal dotted line) were considered positive.

Supplement Figure 6

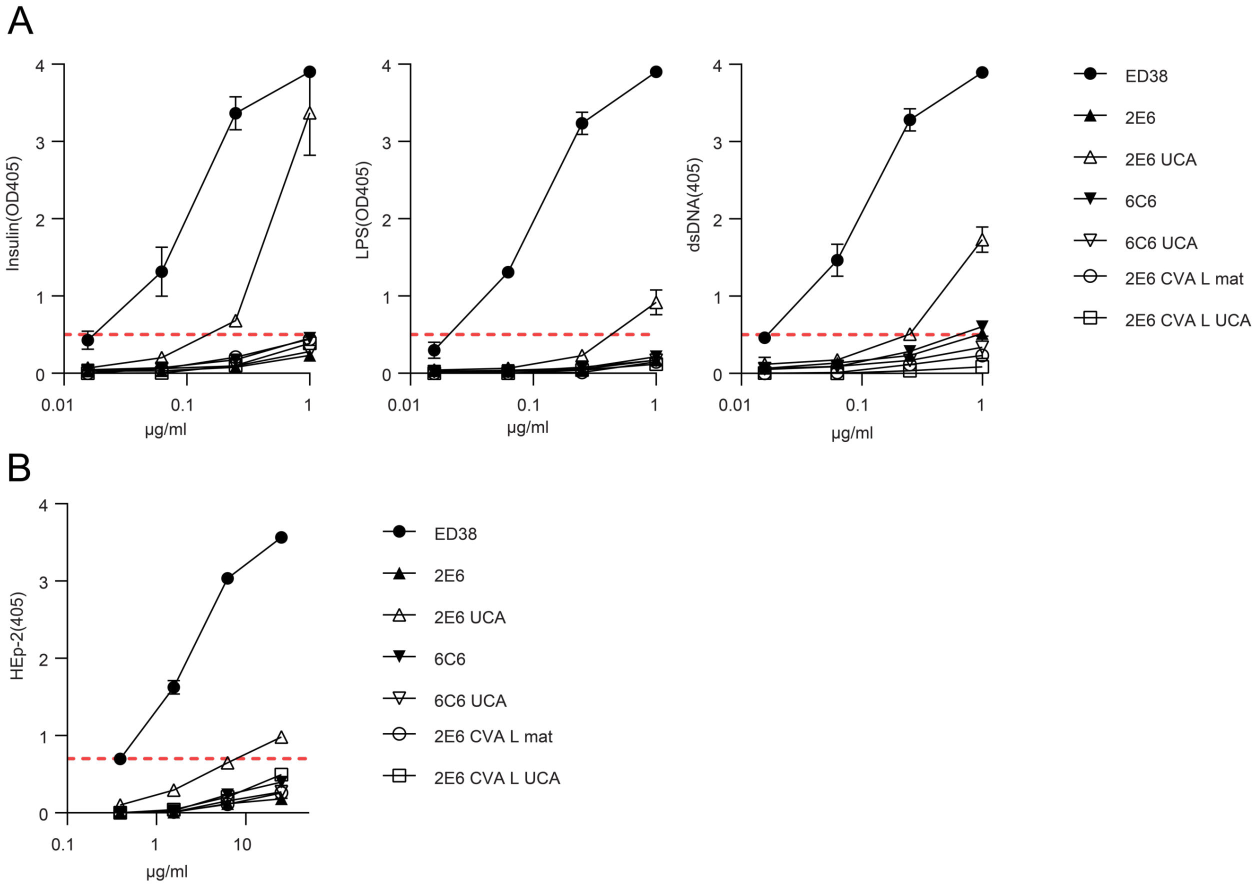

Supplement Figure 6. Polyreactivity and self-reactivity of MuSK mAbs. Polyreactivity and HEp-2 reactivity of MuSK specific mAbs 2E6, 6C6 and their corresponding unmutated common ancestors (UCA). **(a)** The reactivity of the mAbs against LPS, dsDNA and insulin was tested by ELISA. Each data point represents the mean value of two independent experiments and the error bars represent SDs. Dotted horizontal red lines mark the positive reactivity cut-off (0.5) at OD405. **(b)** Purified antibodies were tested for autoreactivity on a solid-phase ELISA against human epithelial type 2 (HEp-2) cell lysate. Antibody reactivity to HEp-2 lysate is illustrated by the binding curves. Each data point represents the mean value of two independent experiments and the error bars represent SDs. Dotted horizontal red lines mark the positive reactivity cut-off (0.7) at OD405. ED38, a monoclonal antibody cloned from a VpreB+L+ peripheral B cell, was used as a positive control for both assays.

Supplement Figure 7

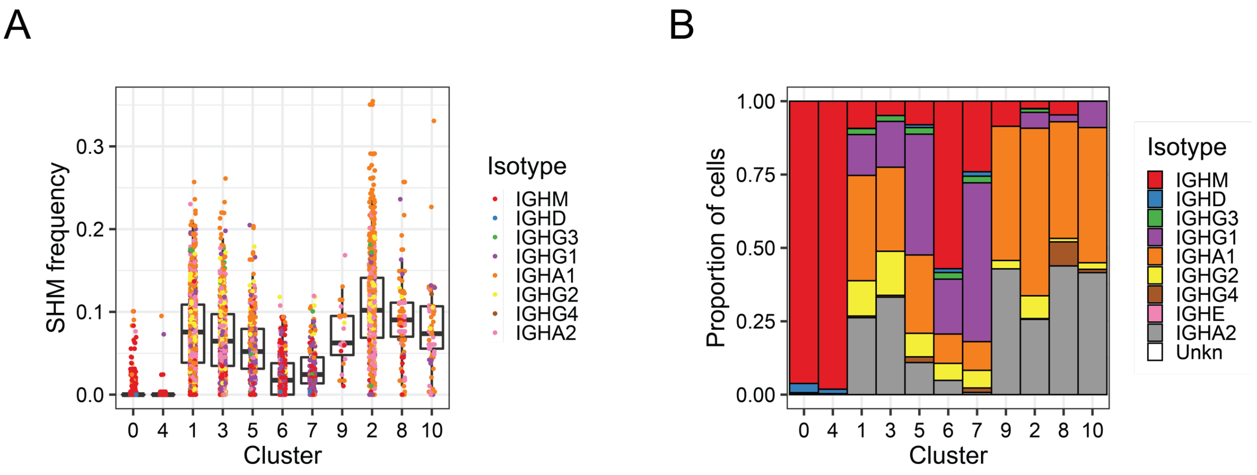

Supplement Figure 7. Somatic mutation frequency and isotype distribution of B cells examined with single cell transcriptional profiling. The frequency of somatic mutations **(a)** and isotype frequency **(b)** of the B cell subset clusters shown in Figure 3 a, b.

Supplement Figure 8

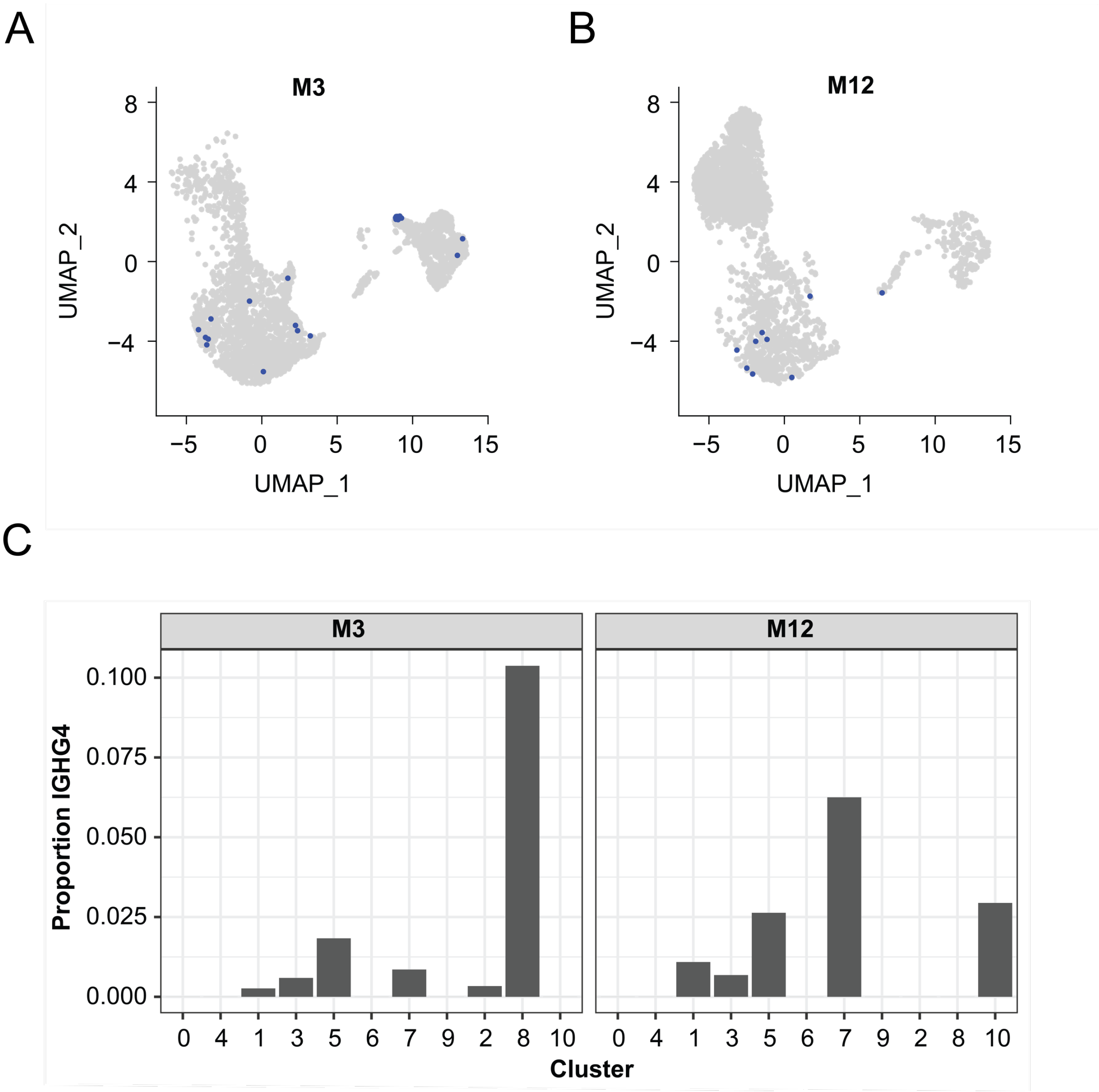

Supplement Figure 8. Distribution of IgG4 expression among B cells clusters identified by single cell transcriptional profiling. (a, b) Manifold Approximation and Projection (UMAP) of B cells obtained at the timepoint 2E6 (**a** (=MuSK MG1 (70 months))) and 6C6 (**b** (=MuSK MG-4 (25 months))) were produced. Each point represents a single cell. B cells expressing IgG4 subclass are indicated with a blue dot. **(c)** Proportion of B cells in each cluster shown in Figure 3 a, b with IgG4 constant regions.

Supplement Figure 9

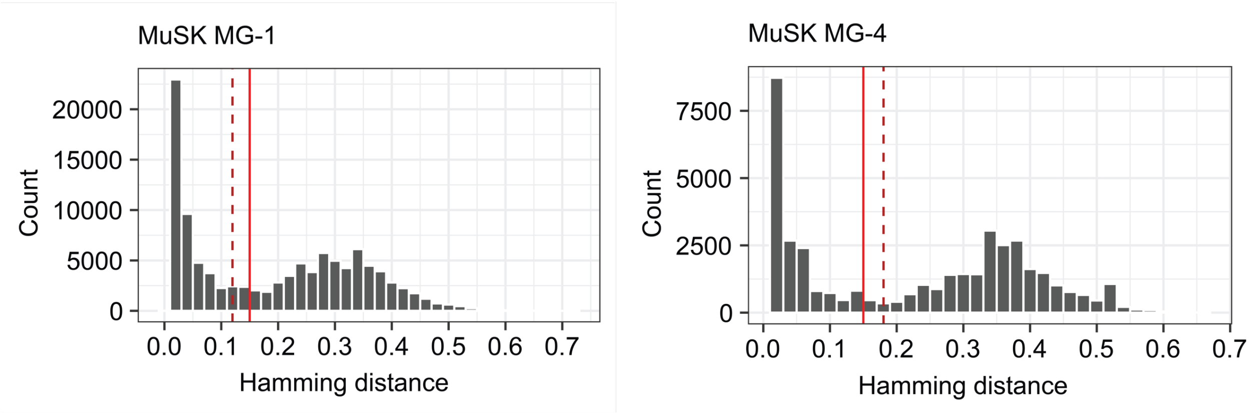

Supplement Figure 9. Distance-to-nearest plots used to identify the threshold required for assigning clonal members in the BCR sequencing data. Distance-to-nearest plots used to identify a common threshold to use for hierarchical clustering-based grouping of V(D)J sequences from high throughout sequencing of BCR repertoires. Red dashed lines correspond to the threshold used for assigning clonal clusters. Grey bars represent the distribution of intra-subject distance-to-nearest distances.

Patient ID	Time (months) since first collection	Sex	Age	Diagnosis	Antibody status	MGFA, MGC class at TOC	Time since last rituximab (years)	Other therapy	Serum MuSK antibody titer
MuSK MG-1	70	F	65	Generalized MuSK MG	MuSK	IIb; 7	1.8	Pred 5 mg/day	56.0
MuSK MG-2	105	F	40	Generalized MuSK MG	MuSK	IIb; 2	1.5	None	12.7
MuSK MG-3	47	F	55	Generalized MuSK MG	MuSK	IIb; 4	0.2	Pred 10 mg/day	0.15
MuSK MG-4	0	F	41	Generalized MuSK MG	MuSK	IIIb; 16	-	Pred 35 mg/day; IVIg 1g/kg q3wks	27.3
MuSK MG-4	25	F	43	Generalized MuSK MG	MuSK	IIIb; 13	1.5	None	16.5
MuSK MG-5	3	F	31	Generalized MuSK MG	MuSK	IIB; 7	-	None	2.01
MuSK MG-5	7	F	31	Generalized MuSK MG	MuSK	IIb; 6	-	PLEX	8.54
MuSK MG-6	0	M	68	Generalized MuSK MG	MuSK	IIIa; 15	-	Pred 60 mg/day; AZA 150 mg/day; Mestinon 60 mg TID; Plex last in Oct 2014	1:320*
MuSK MG-6	16	M	70	Generalized MuSK MG	MuSK	IIb; 8	0.8	Pred 10 mg/day; AZA 150 mg/day	0.03
MuSK MG-7	35	F	36	Generalized MuSK MG	MuSK	IIb; 4	0.9	MMF 1000 mg BD	0.89
MuSK MG-8	94	M	76	Generalized MuSK MG	MuSK	I; 1	7.8	None	0.0
MuSK MG-9	0	F	32	Generalized MuSK MG	MuSK	IIb; 5	-	Pred 40 mg qd	1.75

Supplement Table 1. Study subject clinical, laboratory, and demographic data. MuSK MG patients (n=9 unique patients, n=12 different sample collections) from whom samples were used for isolation of MuSK-specific B cells through single cell sorting followed by B cell culturing. Antibody titer was either measured by Athena Diagnostics (unit = fold dilution) or at the Mayo Clinic Laboratory (unit = nmol/L). Samples measured at Athena Diagnostics are indicated by an (*). The reference range for positivity varies according to the measuring facility. For samples measured by Athena Diagnostics the titer range is negative for <1:10, borderline for 1:10 and positive for >1:20. The cut off for negativity for samples measured at Mayo Clinic Laboratory is ≤ 0.02 nmol/L. TOC = time of collection; Pred = Prednisone; PLEX = plasma exchange; AZA = azathioprine; MMF = Mycophenolate mofetil.

mAb	AChR (CPM)	AChR (ΔCPM)	MuSK (CPM)	MuSK (ΔCPM)	Result
HC1	916	153	51	-6	neg
HC2	494	-269	62	5	neg
HC3	878	115	59	2	neg
AChR PC1	15908	15145	81	24	AChR+
AChR PC2	18913	18150	73	16	AChR+
MuSK PC1	967	204	8916	8859	MuSK +
MuSK PC2	994	231	8712	8655	MuSK +
mAb 2E6	667	-96	8268	8211	MuSK +
mAb 6C6	710	-53	7211	7154	MuSK +

Supplement Table 2. Radioimmunoassay-based testing of the 2E6 and 6C6 mAbs. The radioimmunoassay RIAs were carried out as per manufacturer's instructions. Serum (5 μl) or 5 μl mAb (1 μg/μl) was incubated with 50 μl ^{125}I-MuSK overnight at 4°C. Carrier was added to mAb tubes followed by 50 μL of anti-human IgG to all tubes for 1 hr. The complex was precipitated by centrifugation, washed, and counted for 1 min on a gamma counter. CPM=counts per minute, ΔCPM=CPM-background, HC=healthy control, PC=positive control.

Patient ID	Time Point in Months	Diagnosis	Antibody status	MGFA, MGC at TOC	Time since last Rituximab in months	Other therapy	Serum MuSK antibody titer	Sequencing Data available	Origin of Clone or clonal variant
MuSK MG-1	0	Generalized MuSK MG	MuSK	IIb; 3	28	-	1:2560	+	-
MuSK MG-1	18	Generalized MuSK MG	MuSK	IIb; 5	17	-	1:2560	-	-
MuSK MG-1	19	Generalized MuSK MG	MuSK	0; 0	1	-	1:640	-	-
MuSK MG-1	27	Generalized MuSK MG	MuSK	0; 0	9	-	1:80	-	-
MuSK MG-1	32	Generalized MuSK MG	MuSK	0; 0	14	-	1:1260	+	-
MuSK MG-1	37	Generalized MuSK MG	MuSK	0; 0	19	-	1:5120	+	2E6_CVA
MuSK MG-1	39	Generalized MuSK MG	MuSK	IIb; 7	21	-	1:5120	+	-
MuSK MG-1	45	Generalized MuSK MG	MuSK	0; 0	5	-	1:320	-	-
MuSK MG-1	51	Generalized MuSK MG	MuSK	0; 0	1	-	1:160	-	-
MuSK MG-1	61	Generalized MuSK MG	MuSK	0; 0	11	-	1:320	+	-
MuSK MG-1	68	Generalized MuSK MG	MuSK	0; 0	18	-	1:2560	+	2E6_CVB
MuSK MG-1	70	Generalized MuSK MG	MuSK	IIb; 7	20	Pred 5 mg/day	1:2560	+	2E6 2E6_CVC
MuSK MG-1	79	Generalized MuSK MG	MuSK	0; 0	2	-	1:320	-	-
MuSK MG-4	0	Generalized MuSK MG	MuSK	IIIb; 16	Pre-rituximab	Pred 35 mg/day; IVIg	27.3*	+	-
MuSK MG-4	4	Generalized MuSK MG	MuSK	IIb; 5	2	Pred 15 mg/day	25.0*	-	-
MuSK MG-4	25	Generalized MuSK MG	MuSK	IIIb; 13	18	-	16.5*	+	6C6

Supplement Table 3. Characteristics and analysis status of serial samples from patient MuSK MG-1 and MuSK MG-4. These longitudinally collected samples were used for investigating whether clones or clonal variants of mAbs 6C6 and 2E6 were present. Antibody titer was either measured by CBA in our laboratory or at the Mayo Clinic Laboratory. Samples of MuSK MG-4 measured at Mayo Clinic Laboratory are indicated by an (*); the unit is nmol/L. The cut off for negativity for samples measured at Mayo Clinic Laboratory is ≤ 0.02 nmol/L. The autoantibody titers of MuSK MG-1 measured by our CBA was performed using 10 two-fold dilutions ranging from 1:20 to 1:10240. TOC = time of collection; Pred = Prednisone

Patient ID	Time Point in Months	Source	Reads	IGHA	IGHD	IGHE	IGHG	IGHM	Clones
MuSK MG-1	0	Mandel-Brehm et al. 2021	7666	1514	498	1	529	3707	5319
MuSK MG-1	32	Mandel-Brehm et al. 2021	16117	5559	1811	2	1416	2697	5612
MuSK MG-1	37	Mandel-Brehm et al. 2021	15117	2102	930	0	699	8459	10235
MuSK MG-1	39	Mandel-Brehm et al. 2021	24834	3770	2675	0	1080	11571	15822
MuSK MG-1	61	PacBio	9912	2878	0	0	1111	0	2150
MuSK MG-1	68	NEB	3362	1687	91	0	486	544	1537
MuSK MG-1	68	PacBio	10659	3086	0	0	1106	0	2736
MuSK MG-1	70	10x	3581	1575	11	0	711	446	2218
MuSK MG-1	70	NEB	5476	2697	134	1	694	744	2247
MuSK MG-1	70	PacBio	10526	2815	0	0	1801	0	3072
MuSK MG-4	0	NEB	997	379	19	3	311	125	632
MuSK MG-4	0	PacBio	17059	2955	0	0	1808	0	3995
MuSK MG-4	25	10x	3669	499	78	0	329	2576	3434
MuSK MG-4	25	NEB	4994	1188	353	1	992	1448	2711
MuSK MG-4	25	PacBio	15162	1924	0	0	1345	0	2257

Supplement Table 4. Counts of reconstructed V(D)J sequences by isotype and clones from sequencing of bulk BCR repertoires and single cell transcriptional profiling.
